# Simultaneous inference of haplotypes and alleles at a causal gene

**DOI:** 10.3389/fgene.2015.00291

**Published:** 2015-10-06

**Authors:** Fabrice Larribe, Mathieu J. Dupont, Gabrielle Boucher

**Affiliations:** ^1^Département de Mathématiques, Université du Québec à MontréalMontréal, QC, Canada; ^2^Département d'informatique et de recherche opérationnelle, Université de MontréalMontréal, QC, Canada; ^3^Montreal Heart InstituteMontréal, QC, Canada

**Keywords:** EM algorithm, linkage disequilibrium, causal allele inference, haplotype estimation

## Abstract

We present a methodology which jointly infers haplotypes and the causal alleles at a gene influencing a given trait. Often in human genetic studies, the available data consists of genotypes (series of genetic markers along the chromosomes) and a phenotype. However, for many genetic analyses, one needs haplotypes instead of genotypes. Our methodology is not only able to estimate haplotypes conditionally on the disease status, but is also able to infer the alleles at the unknown disease locus. Some applications of our methodology are in genetic mapping and in genetic counseling.

## 1. Introduction

**Figure 1:**
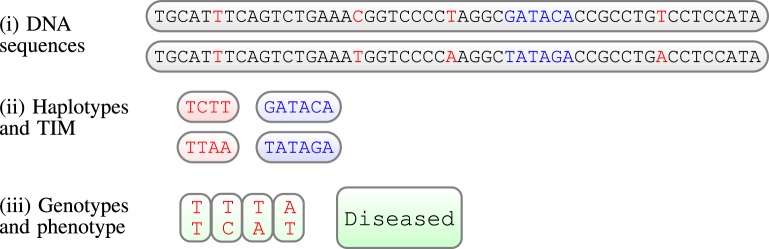
**llustration of a (very short) piece of chromosome (52 base pairs) for one individual; from top to bottom: (i) the unobserved raw data are the 2 DNA sequences; genetic markers in red, TIM in blue; (ii) haplotypes obtained by considering only genetic variations in DNA, and the unobserved TIM; (iii) finally, the observed data: a set of genotypes and a phenotype, in this case a status of a diseased individual**.

In human genetic studies, the unobserved raw data consists of two DNA sequences for each individual (see Figure 1i). As we usually observe few variations along the sequences for different people, we consider only sites which are different between individuals, the genetic markers. In this work we will consider bi-allelic markers, i.e., markers having two possible variations (alleles); the data can then be summarized as a binary couple. A series of genetic markers along the sequence is a haplotype; however, we observe genotypes, not haplotypes. In short, genotypes contain the same information as haplotypes, with the exception that they do not provide the phase information, i.e., we do not know which allele is located on which chromosome (see Figure 1, for an illustration). Moreover, we assume here that the phenotype (indicated by case/control) is caused or influenced by a binary (present or absent) Trait Influencing Mutation (TIM). The position of this TIM is always unknown; obviously, inferring the position of such a TIM is the precise goal of genetic mapping: to infer the location of such a TIM. In Figure 1, the TIM is illustrated as a DNA sequence of length six, however, in general TIMs can be sequences of any length, a single site for example.

For many genetic analyses however, the haplotype data is required, and in some cases even this information may not suffice. The unknown allelic state at the TIM is also required and this is an issue addressed by our work. Indeed, many methodologies use genealogies to infer parameters of populations (such as recombination rate and mutation rate) and these genealogies must be built using haplotypes, not genotypes, since the haplotypes contain the additional information about which genetic material was transmitted from one ancestor to a child. All such methodologies have a way to deal with this problem: some include an estimate of the haplotypes, like the Margarita program (Minichiello and Durbin, 2006), and others defer the issue to external methodologies, like TreeLD (Zöllner and Pritchard, 2005). We introduced an approach for fine genetic mapping using the coalescent with recombination (Larribe et al., 2002), which has the particularity to be the only methodology using genealogies built conditionally on the alleles at the TIM. Of course, as the location of the TIM is unknown, the alleles at the TIM are unknown as well, and hence must be inferred from the data. Finally, the estimates of the alleles at the TIM opens new avenues to evaluate risks associated with the disease: under a standard genetic model, the risk of the disease is a function of the alleles at the TIM.

## 2. Existing methodologies

To our knowledge, none of the current methodologies is able to jointly estimate haplotypes and the alleles at a causal gene. To infer haplotypes from genotypes, laboratory or computational methods can be used (Browning and Browning, 2011). The first statistical method to estimate haplotypes was the parsimony principle proposed by Clark (1990): assuming that the recombination rate is low, haplotypes are inferred by supposing that the number of distinct haplotypes is small. This method is limited to a few markers and works for short sequences. Since then, different methods have used the EM algorithm, for example Excoffier and Slatkin (1995) or Qin et al. (2002). If we wish to estimate the distribution of the haplotypes, we are in an incomplete data setting, and the EM algorithm is a natural solution. Indeed, this algorithm has often been used in this context; for example, one of the earliest methods to use haplotypes frequencies estimated by the EM algorithm is Fallin et al. (2001). One of the more advanced methods to estimate haplotypes is Phase (Stephens et al., 2001; Stephens and Donnelly, 2003); this bayesian method is based on Gibbs sampling and uses coalescent theory to derive the prior distribution of haplotypes. Other methods use information from reference haplotypes to improve phase estimation and infer missing genotypes. A comparison of the efficiency of different methods has been realized by Marchini et al. (2006) and Browning and Browning (2011), provide a recent review of methods that infer haplotypes.

Most of the aforementioned methodologies do not take into account the phenotype, nor the genetic model. In our context of a case/control study, one would not want to ignore the phenotype information. Unlike our method, none of the existing methodologies proposes to estimate the alleles at the TIM.

Finally, note that recent methodologies for estimating haplotypes use human sequence data, but some parts of the human genome are still difficult to sequence, which can limit the use of these methods; it is important to note that our method can be used on human sequences as well as animal or plant sequences, could also be extended to non diploid organisms.

## 3. The EM algorithm

Note that the EM algorithm presented here, by taking into account the phenotype, is in a way an extension of the work of Excoffier and Slatkin (1995), and is also related to the work presented in chapter 5 of Foulkes (2009).

### 3.1. Complete likelihood and M step

Assume a large population of diploid individuals in Hardy-Weinberg equilibrium, where we can observe a dichotomous trait that depends on at least one Trait Influencing Mutation (TIM). As the phenotype ϕ depends on the TIM through a genetic model, it is actually possible for the trait to be dependent on several TIMs, but we consider only one TIM at a time. Let *V*_0_ denote the distribution of haplotypes among non carriers of the TIM, and *V*_1_ denote the distribution of haplotypes among carriers. Alternatively, carrier haplotypes will be called mutant haplotypes, and non carrier haplotypes, primitive haplotypes. For a given type of haplotype *h* (*h* = 1, …, *H*), *V*_0_(*h*) is the proportion of haplotypes of type *h* among non carrier haplotypes, and similarly *V*_1_(*h*) is the proportion of haplotypes of type *h* among carrier haplotypes. A genetic model *F* = (*f*_0_, *f*_1_, *f*_2_) associated with the TIM we are studying is such that *f*_*i*_ is the probability for an individual to express the trait given that it bears *i* = 0, 1, or 2 copies of the causal mutation. Let *T* be the status of an individual at the TIM, such that *T* ∈ {(0, 0), (1, 1), (0, 1), (1, 0)} represents a non carrier, an homozygote carrier or an heterozygote carrier with the TIM inherited from the father or the mother, respectively. Finally, let *p* be the frequency of carrier haplotypes in the population, and *f* the frequency of the trait we are working on.

Since the population is in Hardy-Weinberg equilibrium, we can easily calculate specific probabilities of the form *P*[ϕ, *T* = (δ_1_, δ_2_)]; the sample spaces for *T* and ϕ being of size four and two respectively, there are eight such probabilities for their combinations. For example, as *p*^2^ is the probability for an individual to be a double carrier, and *f*_2_ the probability for a double carrier to express the trait, the probability for any individual *i* in the population to be a double carrier and to express the trait is simply

(1) $$\begin{array}{l} P\left[\phi_{i}=1,T=\left(1,1\right)\right]\;=\;p^{2}f_{2}. \end{array}$$

The other seven cases are treated similarly (see Table 1, for details).

**Table 1:** **Distributions of alleles at the causal gene in the population**.

	**Case**	**Control**	**Total**
*T* = (0,0)	$f_{0}{\left(1-p\right)}^{2}$	$\left(1-f_{0}\right){\left(1-p\right)}^{2}$	(1 − *p*)^2^
*T* = (0,1)	*f*_1_*p*(1 − *p*)	$\left(1-f_{1}\right)p{\left(1-p\right)}^{2}$	*p*(1 − *p*)^2^
*T* = (1,0)	*f*_1_*p*(1 − *p*)	$\left(1-f_{1}\right)p{\left(1-p\right)}^{2}$	*p*(1 − *p*)^2^
*T* = (1,1)	$f_{2}p^{2}$	$\left(1-f_{2}\right)p^{2}$	*p*^2^
Total	*f*	1 − *f*	1

Let *h*^0^ be a non carrier haplotype of type *h*, *h*^1^ a carrier haplotype of type *h*, and $d_{i}=\left(h_{i_{1}}^{\delta_{1}},h_{i_{2}}^{\delta_{2}}\right)$ the diplotype of individual *i* (*i* = 1, …, *n*), where δ_1_, δ_2_ ∈ {0, 1}. Let *G* be a sample of genotypes from the population, and Φ the associated set of phenotypes. As we need to estimate *V*_0_ and *V*_1_, we are in an incomplete data problem, where the complete data is the set of phenotypes Φ and the set of diplotypes *D*, including the alleles at the causal gene. If the alleles (δ_1_, δ_2_) at the causal gene were known, then the probability of the diplotype $d_{i}=\left(h_{i_{1}}^{\delta_{1}},h_{i_{2}}^{\delta_{2}}\right)$ would only depend on the distributions *V*_0_ and *V*_1_, and we would then have:

$$P\left[\left(h_{i_{1}}^{\delta_{1}},h_{i_{2}}^{\delta_{2}}\right)|V_{0},V_{1}\right]=P\left[T=\left(\delta_{1},\delta_{2}\right)\right]\;V_{\delta_{1}}\left(h_{i_{1}}\right)\;V_{\delta_{2}}\left(h_{i_{2}}\right).$$

Since the phenotype depends on the diplotype only through the causal gene, the joint probability of the diplotype $d_{i}=\left(h_{i_{1}}^{\delta_{1}},h_{i_{2}}^{\delta_{2}}\right)$ and the phenotype is:

(2) $$\begin{array}{l} P\left[\left(h_{i_{1}}^{\delta_{1}},h_{i_{2}}^{\delta_{2}}\right),\phi_{i}|V_{0},V_{1}\right]\\ \;=P\left[\phi_{i}|\left(h_{i_{1}}^{\delta_{1}},h_{i_{2}}^{\delta_{2}}\right)\right]P\left[\left(h_{i_{1}}^{\delta_{1}},h_{i_{2}}^{\delta_{2}}\right)|V_{0},V_{1}\right]\\ \;=P\left[\phi_{i}|T=\left(\delta_{1},\delta_{2}\right)\right]P\left[T=\left(\delta_{1},\delta_{2}\right)\right]\;V_{\delta_{1}}\left(h_{i_{1}}\right)\;V_{\delta_{2}}\left(h_{i_{2}}\right)\\ \;=P\left[\phi_{i},T=\left(\delta_{1},\delta_{2}\right)\right]V_{\delta_{1}}\left(h_{i_{1}}\right)\;V_{\delta_{2}}\left(h_{i_{2}}\right). \end{array}$$

We have previously seen how to calculate probabilities of the form *P*[ϕ, *T* = (δ_1_, δ_2_)] (see for example Equation 1); hence it becomes easy to calculate the above probability for each of the eight combinations of δ_1_, δ_2_ and ϕ; for instance:

$$\begin{array}{l} P\left[\left(h_{i}^{1},h_{j}^{1}\right),\phi =1|V_{0},V_{1}\right]=f_{2}\;\cdot \;p^{2}\;\cdot \;V_{1}(h_{i})\;\cdot \;V_{1}(h_{j}),\\ P\left[\left(h_{i}^{0},h_{j}^{1}\right),\phi =0|V_{0},V_{1}\right]=(1-f_{1})\;\cdot \;p\;\cdot \;(1-p)\cdot \\ \;V_{0}(h_{i})\;\cdot \;V_{1}(h_{j}). \end{array}$$

Because individuals are assumed independent, the likelihood of (*V*_0_, *V*_1_) on the complete data is:

$$\begin{array}{l} L_{c}\left(V_{0},V_{1}\right)=P\left[D,\Phi |V_{0},V_{1}\right]\\ \;=\overset{n}{\underset{i=1}{\prod }}P\left[d_{i},\phi_{i}|V_{0},V_{1}\right]\\ \;=\overset{n}{\underset{i=1}{\prod }}P\left[\left(h_{i_{1}}^{\delta_{i_{1}}},h_{i_{2}}^{\delta_{i_{2}}}\right),\phi_{i}|V_{0},V_{1}\right]\\ \;=\overset{n}{\underset{i=1}{\prod }}P\left[\phi_{i},T=\left(\delta_{i_{1}},\delta_{i_{2}}\right)\right]V_{\delta_{i_{1}}}\left(h_{i_{1}}\right)\;V_{\delta_{i_{2}}}\left(h_{i_{2}}\right). \end{array}$$

Since the probabilities *P*[ϕ_*i*_, *T* = (δ_*i*1_, δ_*i*2_)] do not depend on the distributions *V*_0_ and *V*_1_ but only on the penetrance model *F* and on the frequency *p* of carrier haplotypes, by denoting *K*(*F, p*) a function that depends only on *F* and *p*, we have:

$$\begin{array}{l} L_{c}\left(V_{0},V_{1}\right)=K(F,p)\prod_{i=1}^{n}V_{\delta_{i_{1}}}(h_{i_{1}})\;V_{\delta_{i_{2}}}(h_{i_{2}})\\ \;=K(F,p)\prod_{i=h}^{H}V_{0}{\left(h\right)}^{m_{h^{0}}}\;V_{1}{\left(h\right)}^{m_{h^{1}}}, \end{array}$$

where the last expression is obtained by taking the product over the types of haplotypes instead of individuals, and $m_{h^{0}}$ and $m_{h^{1}}$ are, respectively, the numbers of non carrier and carrier sequences of type *h* in *D*. This likelihood belongs to an exponential family, where the sufficient statistics for *V*_0_ and *V*_1_ are the frequencies $m_{h^{0}}$ and $m_{h^{1}}$. If diplotypes were known, we could obtain the theoretical frequencies from the empirical ones. The diplotypes are not observable, but could be estimated if *V*_0_ and *V*_1_ were known.

Denote the expectation of the sufficient statistics by:

$$\begin{array}{l} m_{h^{\delta }}^{\left(k+1\right)}=E\left[m_{h^{\delta }}|V_{0}^{\left(k\right)},V_{1}^{\left(k\right)},G,\phi \right]. \end{array}$$

We then have to maximize the function:

$$\begin{array}{l} W\left(V_{0},V_{1}|V_{0}^{\left(k\right)},V_{1}^{\left(k\right)}\right)\\ \;=\underset{h}{\sum }\left[m_{h^{0}}^{\left(k+1\right)}\log\left(V_{0}\left(h\right)\right)+m_{h^{1}}^{\left(k+1\right)}\log\left(V_{1}\left(h\right)\right)\right] \end{array}$$

with the constraints $\underset{h}{\sum }V_{0}\left(h\right)=1$ and $\underset{h}{\sum }V_{1}\left(h\right)=1$. By incorporating a Lagrange multiplier for each of the two constraints, we then have to optimize the linear expression :

$$\begin{array}{l} W_{L}\left(V_{0},V_{1}|V_{0}^{\left(k\right)},V_{1}^{\left(k\right)}\right)\\ \;=\underset{h}{\sum }\left[m_{h^{0}}^{\left(k+1\right)}\log\left(V_{0}\left(h\right)\right)+m_{h^{1}}^{\left(k+1\right)}\log\left(V_{1}\left(h\right)\right)\right]\\ \;+\;\lambda_{0}\left(1-\sum_{h}V_{0}\left(h\right)\right)+\;\lambda_{1}\left(1-\sum_{h}V_{1}\left(h\right)\right), \end{array}$$

i.e., we obtain maximum likelihood estimates from the complete data. It can be shown that *W*_*L*_ is a maximum if

$$\begin{array}{l} V_{0}\left(h\right)=\frac{m_{h^{0}}^{\left(k+1\right)}}{\lambda_{0}},\;V_{1}\left(h\right)=\frac{m_{h^{1}}^{\left(k+1\right)}}{\lambda_{1}}. \end{array}$$

Applying the constraints, the *M* step of the algorithm consists in evaluating

$$\begin{array}{l} V_{0}{\left(h\right)}^{\left(k+1\right)}=\frac{m_{h^{0}}^{\left(k+1\right)}}{\sum_{h}m_{h^{0}}^{\left(k+1\right)}},\;V_{1}{\left(h\right)}^{\left(k+1\right)}=\frac{m_{h^{1}}^{\left(k+1\right)}}{\sum_{h}m_{h^{1}}^{\left(k+1\right)}}, \end{array}$$

where $\underset{h}{\sum }m_{h^{0}}^{\left(k+1\right)}$ and $\underset{h}{\sum }m_{h^{1}}^{\left(k+1\right)}$ are, respectively, the expected numbers of non carrier and carrier sequences after iteration *k*.

### 3.2. Conditional expectation and the E step

For now we have seen how to evaluate $V_{0}{\left(h\right)}^{\left(k+1\right)}$ and $V_{1}{\left(h\right)}^{\left(k+1\right)}$ with the haplotypes' frequencies; further we have to evaluate the conditional expectations $m_{h^{\delta }}^{\left(k+1\right)}=E\left[m_{h^{\delta }}|V_{0}^{\left(k\right)},V_{1}^{\left(k\right)},G,\phi \right]$. We have seen in Equation (2) that

$$\begin{array}{l} P\left[\left(h_{i_{1}}^{\delta_{1}},h_{i_{2}}^{\delta_{2}}\right),\phi_{i}|V_{0},V_{1}\right]\\ \;=P\left[\phi_{i},T=\left(\delta_{1},\delta_{2}\right)\right]\;V_{\delta_{1}}\left(h_{i_{1}}\right)\;V_{\delta_{2}}\left(h_{i_{2}}\right), \end{array}$$

which gives, by conditioning on the phenotype:

(3) $$\begin{array}{l} P\left[\left(h_{i_{1}}^{\delta_{1}},h_{i_{2}}^{\delta_{2}}\right),\phi_{i}|V_{0},V_{1}\right]\\ \;=P\left[T=\left(\delta_{1},\delta_{2}\right)|\phi_{i}\right]\;P\left[\phi_{i}\right]\;V_{\delta_{1}}\left(h_{i_{1}}\right)\;V_{\delta_{2}}\left(h_{i_{2}}\right). \end{array}$$

Using *P*[ϕ_*i*_ = 1] = *f*, *P*[ϕ_*i*_ = 0] = 1 − *f* and the probabilities found earlier (see Table 1), it is immediate to obtain the probabilities of *T* given the phenotype, for example:

(4) $$\begin{array}{lll} P\left[T_{i}=\left(0,0\right)|\phi_{i}=1\right] & = & \frac{f_{0}{\left(1-p\right)}^{2}}{f},\\ P\left[T_{i}=\left(0,1\right)|\phi_{i}=0\right] & = & \frac{\left(1-f_{1}\right)p{\left(1-p\right)}^{2}}{1-f}. \end{array}$$

The joint probability of the genotype *g*_*i*_ and the phenotype ϕ_*i*_ is obtained from Equation (3) by summing over all the possible diplotypes:

(5) $$\begin{array}{l} P\left[g_{i},\phi_{i}|V_{0},V_{1}\right]\\ \;=\underset{\left(h_{i_{1}}^{\delta_{1}},\;h_{i_{2}}^{\delta_{2}}\right)\in \;g}{\sum }P\left[\left(h_{i_{1}}^{\delta_{1}},h_{i_{2}}^{\delta_{2}}\right),\phi_{i}|V_{0},V_{1}\right]\\ \;=P\left[\phi_{i}\right]\underset{\left(h_{i_{1}}^{\delta_{1}},\;h_{i_{2}}^{\delta_{2}}\right)\in \;g}{\sum }P\left[T=\left(\delta_{1},\delta_{2}\right)|\phi_{i}\right]V_{\delta_{1}}\left(h_{i_{1}}\right)\;V_{\delta_{2}}\left(h_{i_{2}}\right). \end{array}$$

Then, the probability of a diplotype $\left(h_{i_{1}}^{\delta_{1}},h_{i_{2}}^{\delta_{2}}\right)$, given the phenotype ϕ_*i*_ and the genotype *g*_*i*_, is, using Equations (3) and (5):

(6) $$\begin{array}{l} P\left[\left(h_{i_{1}}^{\delta_{1}},h_{i_{2}}^{\delta_{2}}\right)|g_{i},\phi_{i},V_{0},V_{1}\right]\\ \;=\frac{P\left[T=\left(\delta_{1},\delta_{2}\right)|\phi_{i}\right]\;V_{\delta_{1}}(h_{i_{1}})\;V_{\delta_{2}}(h_{i_{2}})}{\sum_{\left(h_{i_{1}}^{\beta_{1}},\;h_{i_{2}}^{\beta_{2}}\right)\in \;g}P\left[T=\left(\beta_{1},\beta_{2}\right)|\phi_{i}\right]\;V_{\beta_{1}}(h_{i_{1}})\;V_{\beta_{2}}(h_{i_{2}})}. \end{array}$$

We can see that the conditional probability depends only on the distributions *V*_0_ and *V*_1_, and the probabilities *P*[*T* ∣ ϕ_*i*_].

Let's now evaluate the conditional expectation $m_{h^{\delta }}^{\left(k+1\right)}$. Let *n*_*g*, ϕ_ be the number of individuals with genotype *g* and phenotype ϕ; among these individuals,

$$n_{g,\phi }\cdot P\left[\left(h^{\delta },\cdot \right)|g,\phi ,|V_{0},V_{1}\right]$$

will receive the sequence *h*^δ^ from their mother, and

$$n_{g,\phi }\cdot P\left[\left(\cdot ,h^{\delta }\right)|g,\phi ,|V_{0},V_{1}\right]$$

from their father. As usual, the conditional probability of having a given sequence as the maternal haplotype is obtained by summing on all the compatible paternal haplotypes (and vice versa). Recall that if there is no missing information on the genotypes, there is a unique sequence *h*_*g*_ compatible with *h* such that (*h, h*_*g*_) ∈ *g*. In this case:

$$\begin{array}{l} P\left[\left(h^{\delta },\cdot \right)|g,\phi ,V_{0},V_{1}\right]=P\left[\left(h^{\delta },h_{g}^{0}\right)|g,\phi ,V_{0},V_{1}\right]\\ \;+P\left[\left(h^{\delta },h_{g}^{1}\right)|g,\phi ,V_{0},V_{1}\right],\\ P\left[\left(\cdot ,h^{\delta }\right)|g,\phi ,V_{0},V_{1}\right]=P\left[\left(h_{g}^{0},h^{\delta }\right)|g,\phi ,V_{0},V_{1}\right]\\ \;+P\left[\left(h_{g}^{1},h^{\delta }\right)|g,\phi ,V_{0},V_{1}\right]. \end{array}$$

These two probabilities being equal by symmetry, the mean number of copies of *h*^δ^ carried by the *n*_*g*, ϕ_ individuals presenting this profile is

$$2\cdot n_{g,\phi }\cdot P\left[\left(h_{g},\cdot \right)|g,\phi ,V_{0},V_{1}\right].$$

We then obtain $m_{h_{\delta }}^{\left(k+1\right)}$ by summing over all the genotypes and phenotypes. The E step of the algorithm reduces to evaluating:

(7) $$\begin{array}{l} m_{h^{\delta }}^{\left(k+1\right)}=\underset{\left(g,\phi \right)\in \left(G,\Phi \right)}{\sum }2\cdot n_{g,\phi }\cdot P\left[\left(h_{g},\cdot \right)|g,\phi ,V_{0},V_{1}\right]. \end{array}$$

for each *h*^δ^. Note that the method can be generalized to missing data, by considering every combination of haplotypes compatible with the observed genotypes.

### 3.3. Non random sampling

We have assumed until now that the sample was obtained by simple random sampling from the population, but usually this is not the case in genetics, since most samples are obtained using a case/control design. Let *n*_1_ be the number of cases in the sample of size *n*, and let ω = *n*_1_ ∕ *n* be the proportion of cases (if the sample were a simple random sample, then ω is expected to be *P*[ϕ] = *f*). In the case/control setting, many methods which estimate haplotypes, including those which use the EM algorithm, are biased since the case/control mode of sampling modifies the distributions of alleles and haplotypes.

In this section we show that the algorithm described in Section 2.2 is robust to this case/control sampling. The proportions given in Table 1, as well as the penetrance model, are not affected by the sampling. Let's see in a first step the behavior of some probabilities under this stratified sampling design. The probabilities of *T* conditional on the phenotype do not change, i.e., *P*[*T* ∣ ϕ, *n*_1_] = *P*[*T* ∣ ϕ], and the probabilities defined previously are still valid. Expected distributions are then easily obtained (see Table 2).

**Table 2:** **Distributions of alleles at the causal gene in the population in a sample with a fixed proportion of cases**.

	**Case**	**Control**	**Total**
*T* = (0,0)	$f_{0}{\left(1-p\right)}^{2}\cdot \frac{\omega }{f}$	$\left(1-f_{0}\right){\left(1-p\right)}^{2}\cdot \frac{\left(1-\omega \right)}{\left(1-f\right)}$	*q*_00_
*T* = (0,1)	$f_{1}p\left(1-p\right)\cdot \frac{\omega }{f}$	$\left(1-f_{1}\right)p{\left(1-p\right)}^{2}\cdot \frac{\left(1-\omega \right)}{\left(1-f\right)}$	*q*_01_
*T* = (1,0)	$f_{1}p\left(1-p\right)\cdot \frac{\omega }{f}$	$\left(1-f_{1}\right)p{\left(1-p\right)}^{2}\cdot \frac{\left(1-\omega \right)}{\left(1-f\right)}$	*q*_10_
*T* = (1,1)	$f_{2}p^{2}\cdot \frac{\omega }{f}$	$\left(1-f_{2}\right)p^{2}\cdot \frac{\left(1-\omega \right)}{\left(1-f\right)}$	*q*_11_
Total	ω	1 − ω	1

Let's now review the steps of the algorithm. The likelihood on the complete data for such a stratified sample, conditional on the number of cases, is:

$$\begin{array}{l} L_{c}\left(V_{0},V_{1}\right)=P\left[D,\Phi |V_{0},V_{1},n_{1}\right]\\ \;=\frac{P\left[D,\Phi ,V_{0},V_{1},n_{1}\right]}{P\left[n_{1}|V_{0},V_{1},n_{1}\right]\;P\left[V_{0},V_{1}\right]}\\ \;=\frac{P\left[D,\Phi ,n_{1}|V_{0},V_{1}\right]}{P\left[n_{1}|V_{0},V_{1},n_{1}\right]\;P\left[V_{0},V_{1}\right]}. \end{array}$$

Since knowledge of the phenotypes Φ carries knowledge of *n*_1_, *n*_1_ can be removed from the numerator. Moreover, the probability of obtaining *n*_1_ cases from a simple random sample does not depend on the distributions *V*_0_ and *V*_1_. After removing terms which do not depend on *V*_0_ and *V*_1_, the likelihood for this data is the same as before:

$$\begin{array}{l} L_{c}\left(V_{0},V_{1}\right)=\frac{P\left[D,\Phi ,n_{1}|V_{0},V_{1}\right]}{P\left[n_{1}\right]}\\ \;=\frac{P\left[D,\Phi |V_{0},V_{1}\right]}{\left(\begin{array}{c} n\\ n_{1} \end{array}\right)f^{n^{1}}{\left(1-f\right)}^{n-n_{1}}}\\ \;=K\left(F,p,n_{1}\right)\prod_{h}V_{0}{\left(h\right)}^{m_{h^{0}}}\;V_{1}{\left(h\right)}^{m_{h^{1}}}, \end{array}$$

which shows the likelihood remains the same for case/control sampling, and hence the M step of the algorithm remains unchanged.

Recall that the E step depended on diplotypes' probabilities, conditional on the genotype and the phenotype. We prove that these probabilities are not modified by the type of sampling. Let's begin by calculating the joint probability of a diplotype and a phenotype, conditional on ω, the proportion of cases; this probability is obtained by adding a condition on the proportion of cases in Equation (3):

$$\begin{array}{l} P\left[\left(h_{i_{1}}^{\delta_{1}},h_{i_{2}}^{\delta_{2}}\right),\phi_{i}|V_{0},V_{1},\omega \right]\\ \;=P\left[\phi_{i}|n_{1}\right]P\left[T=\left(\delta_{1},\delta_{2}\right)|\phi_{i},\omega \right]\\ \;\times \;P\left[\left(h_{i_{1}},h_{i_{2}}\right)|T=\left(\delta_{1},\delta_{2}\right),\omega \right]. \end{array}$$

Once the status at the causal gene is determined, the diplotype probability depends only on the distributions *V*_0_ and *V*_1_. Moreover, if the phenotype is known, *T* does not depend on ω; only the probability of the phenotype is modified:

$$\begin{array}{l} P\left[\left(h_{i_{1}}^{\delta_{1}},h_{i_{2}}^{\delta_{2}}\right),\phi_{i}|V_{0},V_{1},\omega \right]\\ \;=P\left[\phi_{i}|\omega \right]P\left[T=\left(\delta_{1},\delta_{2}\right)|\phi_{i}\right]V_{\delta_{1}}\left(h_{i_{1}}\right)\;V_{\delta_{2}}\left(h_{i_{2}}\right). \end{array}$$

Following the derivation for a simple random sample, the term *P*[ϕ_*i*_ ∣ ω] cancels out in the conditional probability formula, and we get the same result as before. Because these probabilities are not affected by the sampling design, the E step of the algorithm, described in Equation (8) remains the same. We have shown that this EM algorithm can be applied to case/control samples.

### 3.4. Overview of the algorithm

Assume the penetrance model *F* = (*f*_0_, *f*_1_, *f*_2_) and the frequency of the causal mutation are known. The steps of our algorithm are:

1. Compute the probabilities *P*[*T* = (*i, j*) ∣ ϕ = δ] (see Equation 4), for (*i, j*, δ) ∈ {0, 1};
2. Consider an initial $V_{0}^{\left(0\right)}$ and $V_{1}^{\left(0\right)}$ probability distribution (in absence of a priori information on the frequency of haplotypes, we use a uniform distribution);
3. E Step:
  - For each genotype *g* and phenotype ϕ (see Equation 3):
    (1) Evaluate, for all $\left[h_{i_{1}}^{\delta_{1}},h_{i_{2}}^{\delta_{2}}\right]\in g$:
      $$\begin{array}{l} P\left[\left(h_{i_{1}}^{\delta_{1}},h_{i_{2}}^{\delta_{2}}\right),\phi_{i}|V_{0}^{\left(k\right)},V_{1}^{\left(k\right)}\right]\;\\ \;\propto V_{\delta_{1}}^{\left(k\right)}\left(h_{i_{1}}\right)\;V_{\delta_{2}}^{\left(k\right)}\left(h_{i_{2}}\right)\;P\left[T=\delta_{1}\delta_{2}|\phi \right] \end{array}$$
    (2) Sum these probabilities to obtain:
      $$\begin{array}{l} P\left[g,\phi_{i}|V_{0}^{\left(k\right)},V_{1}^{\left(k\right)}\right]\\ \;=\underset{\left(h_{i_{1}}^{\delta_{1}},\;h_{i_{2}}^{\delta_{2}}\right)\in \;g}{\sum }P\left[\left(h_{i_{1}}^{\delta_{1}},h_{i_{2}}^{\delta_{2}}\right),\phi |V_{0}^{\left(k\right)},V_{1}^{\left(k\right)}\right]. \end{array}$$
    (3) The conditional probability can then be computed as:
      $$\begin{array}{l} P\left[\left(h_{i_{1}}^{\delta_{1}},h_{i_{2}}^{\delta_{2}}\right)|g,\phi ,V_{0}^{\left(k\right)},V_{1}^{\left(k\right)}\right]\\ \;=\frac{P\left[\left(h_{i_{1}}^{\delta_{1}},h_{i_{2}}^{\delta_{2}}\right),\phi_{i}|V_{0}^{\left(k\right)},V_{1}^{\left(k\right)}\right]}{P\left[g,\phi |V_{0}^{\left(k\right)},V_{1}^{\left(k\right)}\right]}. \end{array}$$
  - Compute, for each sequence *h*^δ^ (see Equation 8):
    (8) $$\begin{array}{l} m_{h^{\delta }}^{\left(k+1\right)}=\sum_{\left(g,\phi )\in (G,\Phi \right)}2\;\cdot \;n_{g,\phi }\;\cdot \\ \;P\left[\left(h_{g},\;\cdot \right)|g,\phi ,V_{0}^{\left(k\right)},\;V_{1}^{\left(k\right)}\right]. \end{array}$$
4. M Step: update the *V*_·_ distributions, by evaluating, for all *h* (see Equation 3.1):
  $$\begin{array}{l} V_{0}{\left(h\right)}^{\left(k+1\right)}=\frac{m_{h^{0}}^{\left(k+1\right)}}{\sum_{h}m_{h^{0}}^{\left(k+1\right)}},\;V_{1}{\left(h\right)}^{\left(k+1\right)}=\frac{m_{h^{1}}^{\left(k+1\right)}}{\sum_{h}m_{h^{1}}^{\left(k+1\right)}}. \end{array}$$
5. Convergence test. Convergence is reached when
  $$\begin{array}{l} \underset{h,\delta }{\max}|V_{\delta }{\left(h\right)}^{\left(k+1\right)}-V_{\delta }{\left(h\right)}^{\left(k\right)}|<\epsilon . \end{array}$$
  One convergence is reached, let ${\hat{V}}_{0}=V_{0}^{\left(k+1\right)}$ and ${\hat{V}}_{1}=V_{1}^{\left(k+1\right)}$. Otherwise, go back to step 3.

We have assumed that the proportion of carrier haplotypes is known, which is of course not realistic in practice. Note however, by assuming that the penetrance model *F* is known, and that the frequency of the disease *f* is known as well, we can obtain *p*. Since

$$\begin{array}{l} f=f_{0}{\left(1-p\right)}^{2}+2f_{1}p\left(1-p\right)+f_{2}p^{2}, \end{array}$$

if *f*_0_ + *f*_2_ − 2*f*_1_ = 0, then *p* = (*f* − *f*_0_) ∕ 2(*f*_1_ − *f*_0_). In general, however, we have:

$$\begin{array}{l} p=\frac{f_{0}-f_{1}\pm \sqrt{f_{1}^{2}-f_{0}f_{1}+f\left(f_{0}-2f_{1}+f2\right)}}{f_{0}-2f1+f_{2}}, \end{array}$$

and there exists a solution in [0, 1] which satisfies the penetrance model. If 0 ≤ *f*_0_ ≤ *f*_1_ ≤ *f*_2_, then the solution is unique. The methodology has been implemented in C++, and is available from the corresponding author.

**Figure 2:**
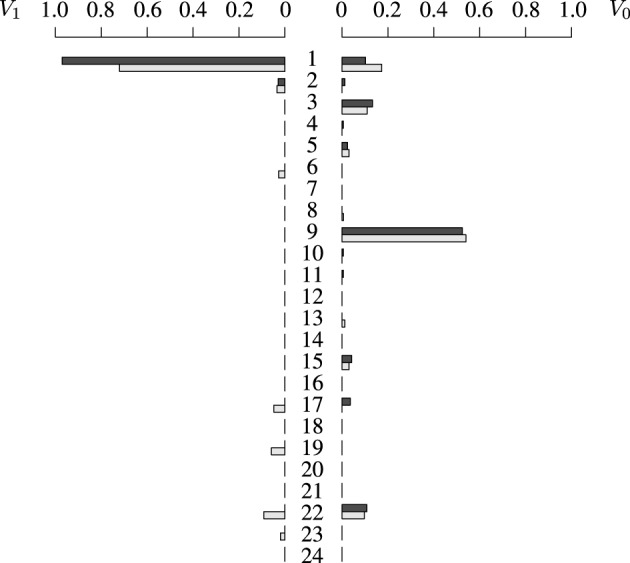
**Distribution of *V*_1_ (left) and *V*_0_ (right)**. Estimated distributions in light gray, and exact distributions in black. Each of the 24 horizontal bars represents a frequency of a particular haplotype type which is compatible with the data: of the 2^8^ possible haplotypes of length 8 markers, only 24 were compatible with the observed genotypes.

For the proposed illustration, we have used ms (Hudson, 2002) to sample 10,000 chromosomes of approximate length 250 kb (simulated using ρ = 100), and randomly assigned pairs of chromosomes to form diploid individuals. One of the markers is chosen randomly such that its minimum allele frequency is approximately 0.10, and this marker will become the TIM. For each individual, a phenotype is then simulated using the two alleles at the TIM according to the genetic model *F* = (0.05, 0.10, 0.80). Haplotypes are then mixed in order to obtain genotypes, and information on haplotypes is discarded. We sample 100 individuals and sequences of length 8 markers, so 2^8^ haplotypes are possible in theory, but only 24 of them are compatible with the observed genotypes. Figure 2 shows the estimates of vectors *V*_0_ and *V*_1_ for each of the 24 possible types of haplotypes in the sample. By comparing individual values, *V*_1_(1) and ${\hat{V}}_{1}\left(1\right)$ for example, we see that the estimates of the frequencies are very good. Note that the estimates of *V*_0_(·) seem to be slightly better than those of *V*_1_(·); this is due to the fact that we have more information on control haplotypes than on case haplotypes, because phenocopy causes many case haplotypes to be non carriers.

## 4. Estimating the causal alleles

The methodology presented in this paper is the first to permit one to jointly estimate the haplotypes at genotyped markers and the (non observed) alleles at the TIM. Estimating the causal alleles could be very useful in genetic counseling for example, where the patient's risk and treatment could be adjusted if the alleles at the disease genes were known. In the sequel, we assume that haplotypes are known and we assess the capacity of our method to infer the causal alleles. As explained in Dupont (2012) or Boucher (2009), when the haplotypes are known, Equation (6) reduces to:

$$\begin{array}{l} P\left[T=\left(\delta_{1},\delta_{2}\right)|d=\left(h_{1},h_{2}\right),\phi ,V_{0},V_{1}\right]\\ =\frac{P\left[T=\left(\delta_{1},\delta_{2}\right)|\phi \right]\;V_{\delta_{1}}\left(h_{1}\right)\;V_{\delta_{2}}\left(h_{2}\right)}{\sum_{T}P\left[T=\left(\delta_{1},\delta_{2}\right)|\phi \right]\;V_{\delta_{1}}\left(h_{1}\right)\;V_{\delta_{2}}\left(h_{2}\right)}. \end{array}$$

In the EM algorithm, the number of parameters increases as the number of genetic markers increases: since the markers are binary, if sequences of length *d* are used, there are 2^*d*^ possible haplotypes, leading to a maximum of 2^*d*^ − 1 parameters to estimate. For this reason, and because huge numbers of genetic markers are available today, the method is illustrated here using a moving windows strategy, i.e., we use windows made of *d* markers each, and the total number of markers is *L* (*d* < *L*). The first window consists of the set of markers {1, 2, …, *d*}, the second consists of the set of markers {2, …, *d* + 1}, and so on.

Let *n*_cas_ and *n*_con_ be the numbers of case and control haplotypes, and *n*^0^ and *n*^1^ the numbers of non carrier and carrier haplotypes, respectively. Let $n_{\text{cas}}^{1}$ and $n_{\text{con}}^{1}$ be the numbers of case and control carrier haplotypes, and $n_{\text{cas}}^{0}$ and $n_{\text{con}}^{0}$ the numbers of case and control non carrier haplotypes, respectively. We then have $n=n^{0}+n^{1}=n_{\text{cas}}+n_{\text{con}}=n_{\text{cas}}^{0}+n_{\text{con}}^{0}+n_{\text{cas}}^{1}+n_{\text{con}}^{1}$. Finally, let $n_{c}^{\delta }\left(0\right)$ and $n_{c}^{\delta }\left(1\right)$ be the numbers of haplotypes, where the *c* subscript denotes the case/control status (*c* ∈ {cas, con}) and δ superscript (δ ∈ {0, 1}) denotes the true non carrier/carrier status of the individuals in the counts $n_{c}^{\delta }\left(0\right)$ and $n_{c}^{\delta }\left(1\right)$. For example, out of a total of $n_{\text{cas}}^{1}=100$ carrier cases, if 75 are correctly estimated as being carriers ($n_{\text{cas}}^{1}\left(1\right)=75$), then 25 are erroneously estimated as being non carriers ($n_{\text{cas}}^{1}\left(0\right)=25$), because $n_{\text{cas}}^{1}\left(1\right)+n_{\text{cas}}^{1}\left(0\right)=n_{\text{cas}}^{1}$. We then define the partial success rates:

$$\begin{array}{lll} \pi_{\text{con}}^{0} & = & \frac{n_{\text{con}}^{0}\left(0\right)}{n_{\text{con}}^{0}},\;\pi_{\text{con}}^{1}=\frac{n_{\text{con}}^{1}\left(1\right)}{n_{\text{con}}^{1}},\\ \pi_{\text{cas}}^{0} & = & \frac{n_{\text{cas}}^{0}\left(0\right)}{n_{\text{cas}}^{0}},\;\pi_{\text{cas}}^{1}=\frac{n_{\text{cas}}^{1}\left(1\right)}{n_{\text{cas}}^{1}}, \end{array}$$

and semi-partial success rates:

$$\begin{array}{lll} \pi_{\text{con}} & = & \frac{n_{\text{con}}^{0}\left(0\right)+n_{\text{con}}^{1}\left(1\right)}{n_{\text{con}}},\;\pi_{\text{cas}}=\frac{n_{\text{cas}}^{0}\left(0\right)+n_{\text{cas}}^{1}\left(1\right)}{n_{\text{cas}}},\\ \pi^{0} & = & \frac{n_{\text{con}}^{0}\left(0\right)+n_{\text{cas}}^{0}\left(0\right)}{n^{0}},\;\pi^{1}=\frac{n_{\text{con}}^{1}\left(1\right)+n_{\text{cas}}^{1}\left(1\right)}{n^{1}}; \end{array}$$

finally, we have the global success rate:

$$\begin{array}{l} \pi =\frac{n_{\text{con}}^{0}\left(0\right)+n_{\text{con}}^{1}\left(1\right)+n_{\text{cas}}^{0}\left(0\right)+n_{\text{cas}}^{1}\left(1\right)}{n_{\text{con}}^{0}+n_{\text{con}}^{1}+n_{\text{cas}}^{0}+n_{\text{cas}}^{1}}=\frac{n^{0}\left(0\right)+n^{1}\left(1\right)}{n}. \end{array}$$

**Figure 3:**
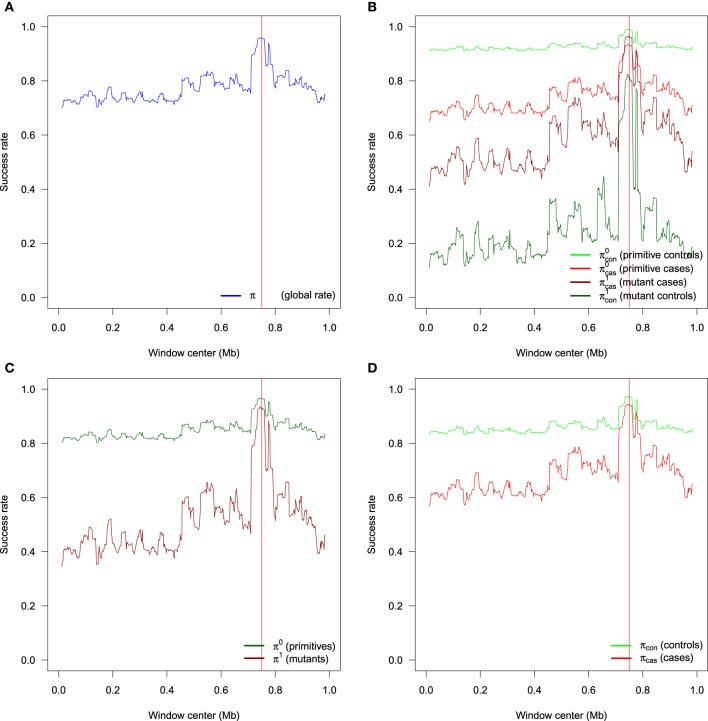
**Example of the different success rates for a particular sample of 400 cases and 400 controls obtained with the genetic model *F* = (0.01, 0.1, 0.1)**. **(A)** Global success rate. **(B)** Partial success rates: primitive controls, primitive cases, mutant cases, mutant controls. **(C)** Semi-partial success rates: primitives and mutants. **(D)** Semi-partial success rates: controls and cases. Results are obtained with windows of width *d* = 16 SNPs incremented by one. There are a total of 500 SNPs.

All these rates have different meanings, and are useful depending on the question of interest. In particular, π^0^ is the probability to estimate a non carrier if the individual is a non carrier, in other word the specificity, while π^1^ is the probability to estimate a carrier if the individual is a carrier, in other word the sensitivity. The probability π is known as the accuracy. As with all classification rules, it is not informative to achieve high sensitivity without specificity and vice versa. Accuracy alone is not an ideal measure of success for low frequency TIM, as high accuracy could be achieved by simply setting *n*_1_ = 0. Figure 3 exhibits an example of the different success rates for a particular sample of 400 cases and 400 controls obtained with the genetic model *F* = (0.01, 0.1, 0.1). Results are obtained with 500 SNPs using windows of width *d* = 16 SNPs incremented by one. The EM algorithm is run for each of the *L* − *d* + 1 windows along the sequence. The different success rates are estimated for each window and plotted at the center of the window. In each window along the sequence, the rate indicates the proportion of haplotypes for which the allele at the TIM is correctly inferred. In all figures, the real position of the TIM is indicated by a red vertical line. We can see in this example that there is variability along the sequences, and this is easier to estimate TIM alleles for primitive controls (light green) than mutant controls (dark green). All rates increase in the vinicity of the TIM; for instance, consider the global rate π (Figure 3A) which ranges from 0.70 to 0.96 at the position of the TIM. The mean global rate along the sequence is 0.77. The increased success rate near the TIM is due to more linkage disequilibrium around the TIM, and the difference in haplotypes between cases and controls is more informative.

The accuracy of our method depends on several factors. We have identified three of them: the genetic model, the sample size, and the windows width *d*. To identify the impact of each of these factors, we present further analyses where we vary the factors one at a time. In order to assess the effect on the estimation of the TIM's allele only, we assume here that the haplotypes are known. The strength of the genetic model will be measured using the risk ratio, RR, which is the ratio of the risk of one specified genotype compared to the genotype with no carrier allele. On a simulated population of 50,000 haplotypes and 10,000 SNPs generated by FastSimCoal (Excoffier et al., 2013), 40 of datasets are produced for various genetic models (RR_1_ = *f*_1_ ∕ *f*_0_ ∈ {1.01, 1.1, 2, 10}, RR_2_ = *f*_2_ ∕ *f*_0_ ∈ {1.01, 1.1, 2, 10}, for different sample sizes (*n*_con_ ∕ *n*_cas_ ∈ {100∕100, 200∕200, 400∕400, 800∕800}) and for various window of widths *d* ∈ {2, 4, 8, 16}. Low relative risks RR_1_ and RR_2_ implies there is less information in the data to infer mutant haplotypes. To obtain an informative range of values for RR_1_ and RR_2_, we fixed *f*_0_ at 0.01 and allowed *f*_1_ and *f*_2_ to take every value in the set {0.0101, 0.011, 0.02, 0.1} such that *f*_0_ ≤ *f*_1_ ≤ *f*_2_. These combinations lead to various genetic models, including recessive and dominant ones. Regarding the windows width, we expect that short windows contain less information about the data, however very large windows can cause many single haplotypes, making *V*^0^ and *V*^1^ difficult to estimate. Each sample originates from the same population, with a TIM frequency of *p* = 0.1. The same 500 selected SNPs are used for all analyses. The disease frequency in the population, is calculated as $f_{0}\cdot {\left(1-p\right)}^{2}+f_{1}\cdot 2\cdot p\left(1-p\right)+f_{2}\cdot p^{2}$, and ranges in values from 0.01 to 0.0271. In a case control design with equal proportions of cases and controls, the frequency of the disease itself will have no effect on the proposed methodologies, but the genetic models will.

**Figure 4:**
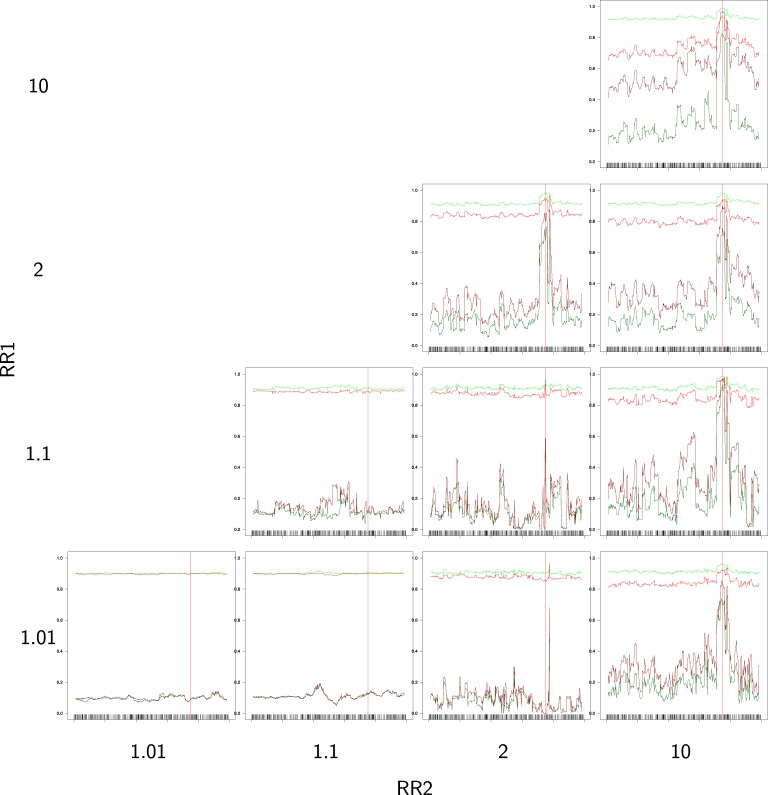
**Partial success rate depending on relative risks RR_1_ = RR_2_ = 10, for a sample size of 400/400 cases/controls, and windows of 16 SNPs**. Scale on *Y* axis: [0, 1]. Light green: primitive controls ($\pi_{\text{con}}^{0}$), red: primitive cases ($\pi_{\text{cas}}^{0}$), dark red: mutant cases ($\pi_{\text{cas}}^{1}$), dark green: mutant controls ($\pi_{\text{con}}^{1}$).

Figure 4 provides the partial success rates for different combinations of RR_1_ and RR_2_. When relative risks are low, we observe that the rates are constant along the sequences, with $\pi_{\text{c}on}^{0}$ and $\pi_{\text{c}as}^{0}$ very high and $\pi_{\text{c}on}^{1}$ and $\pi_{\text{c}as}^{1}$ very low (in fact, these rates are near *p* = 0.1 and 1 − *p* = 0.9, which are the random success rates without any genetic effect). As soon as the relative risk increases, we observe an improvement in the estimation of the causal alleles, this improvement being very noticeable in region of the TIM.

**Figure 5:**
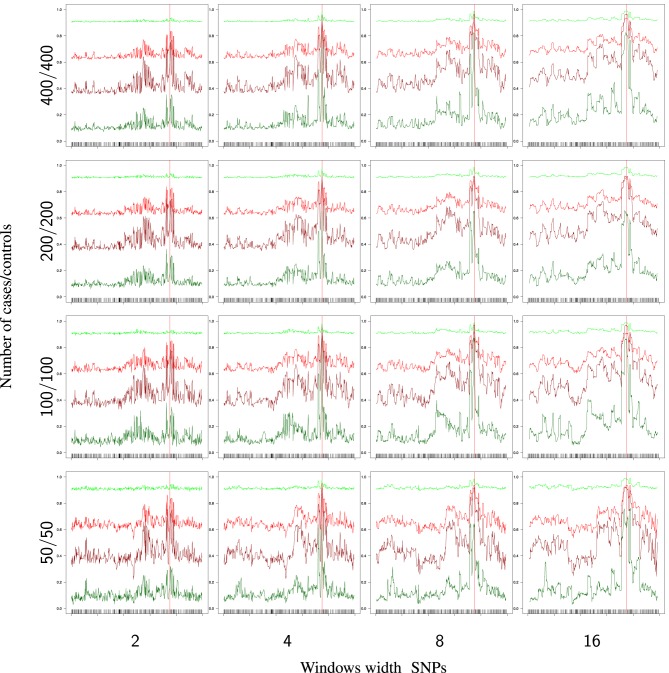
**Partial success rates depending on sample size and windows width for RR_1_ = RR_2_ = 10**. Scale on the *Y* axis: [0, 1]. Light green: primitive controls ($\pi_{\text{con}}^{0}$), red: primitive cases ($\pi_{\text{cas}}^{0}$), dark red: mutant cases ($\pi_{\text{cas}}^{1}$), dark green: mutant controls ($\pi_{\text{con}}^{1}$).

The effect of the windows width (*d*) and the sample size are shown in Figure 5. There is a general improvement in the success rates ($\pi_{\text{con}}^{0}$, $\pi_{\text{cas}}^{0}$, $\pi_{\text{cas}}^{1}$, $\pi_{\text{con}}^{1}$) when these two factors increase, but, surprisingly, the effect is not very strong, perhaps due to the high relative risk assumed in these analyses.

**Figure 6:**
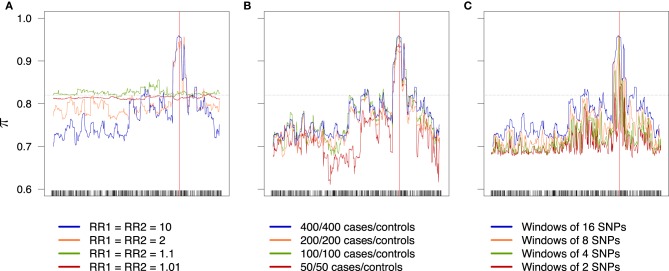
**Global success rates depending on combined relative risks (A), sample size (B), and windows width (C)**. Scale is the same for all three plots. The dotted line represents the random success rate (0.82). **(A)** 400/400 cases/controls, windows of 16 SNPs. **(B)** RR_1_ = RR_2_ = 10, windows of 16 SNPs. **(C)** RR_1_ = RR_2_ = 10, 400/400 cases/controls.

**Figure 7:**
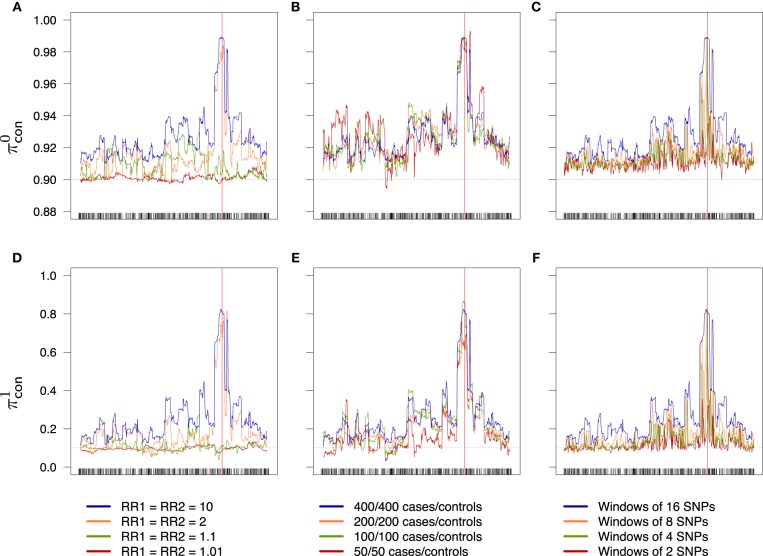
**Success rates of primitive controls (A–C) and mutant controls (D–F) depending on combined relative risks (A,D), sample size (B,E) and windows width (C,F)**. Scale is the same for a given line. Dotted lines represent the random success rate (**A–C**: 0.9; **D–F**: 0.1). **(A,D)** 400/400 cases/controls, windows of 16 SNPs. **(B,E)** RR_1_ = RR_2_ = 10, windows of 16 SNPs. **(C,F)** RR_1_ = RR_2_ = 10, 400/400 cases/controls.

**Figure 8:**
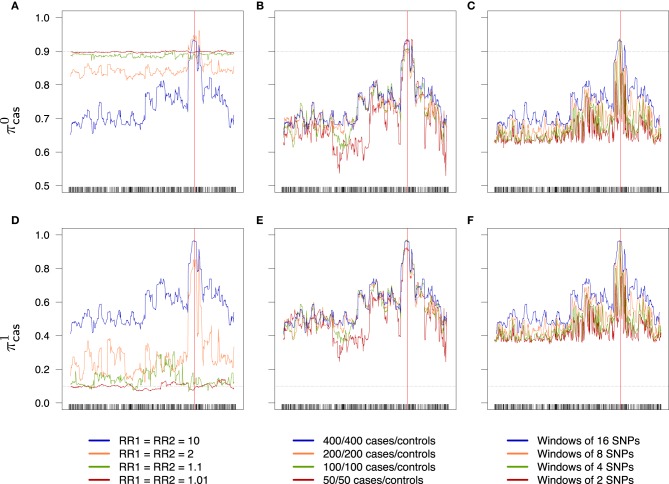
**Success rates of primitives cases (A–C) and mutants cases (D–F) depending on combined relative risks (A,D), sample size (B,E) and windows width (C,F)**. Scale is the same for a given line. Dotted lines represent the random success rate (**A–C**: 0.9; **D–F**: 0.1). **(A,D)** 400/400 cases/controls, windows of 16 SNPs. **(B,E)** RR_1_ = RR_2_ = 10, windows of 16 SNPs. **(C,F)** RR_1_ = RR_2_ = 10, 400/400 cases/controls.

An overview of the effect of the different factors on the global rate π (the accuracy) is shown in Figure 6; an increase in the relative risks clearly translates to an improvement of the global success rate. If the sample size is larger than 50/50 for cases/controls, increasing the sample size seems to have little effect, at least with the parameters considered in this example. Finally, the effect of the windows width is pretty clear: π increases all along the sequence when the width of the windows increases. Similar results are obtained for the partial rates $\pi_{\text{con}}^{1}$, $\pi_{\text{con}}^{0}$ (see Figure 7) and for the partial rates $\pi_{\text{cas}}^{1}$, $\pi_{\text{cas}}^{0}$ (see Figure 8).

**Figure 9:**
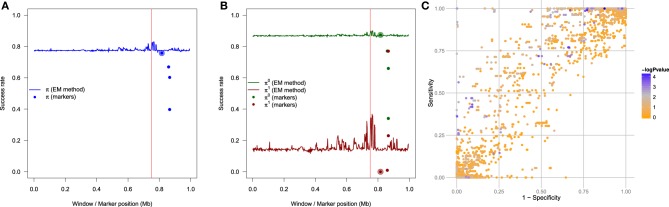
**Using the genetic model RR_1_ = RR_2_ = 2**. **(A)** The blue line indicates the global success rate when the TIM is estimated by our proposed methodology, and the four dots around the position of the TIM indicate the success rate when the TIM is estimated by the four most significant markers (the naive method); the marker showing the strongest association is the largest of the four points. **(B)** The green line indicates the success rate π^0^ (specificity) and the the red line indicates the success rate π^1^ (sensitivity) both obtained with TIM estimated by our proposed methodology; the four green dots are the specificity of the four most associated markers (naive method), and the four red dots are the sensitivity of the same four markers (naive method). **(C)** Relation between sensitivity and 1-specificity, showing −log_10_ of the *p*-value for each marker.

We have compared this EM methodology to a simpler naive method, which consists of testing the association of each marker in the region with the phenotype, and to infer the alleles at the TIM to be the alleles at the marker having the strongest association with the phenotype. To illustrate this procedure, we have used 7503 heterozygote markers to test the association on the data in the case RR_1_ = RR_2_ = 2. As shown in Figure 9A, for each of the four markers showing the strongest association with the phenotype, we have inferred the TIM's alleles to be the alleles of this marker, and plotted the success rate π at the position of these markers. One can see that the success rate for the naive method is lower than the success rate of the EM method around the true position of the TIM, which is expected since the EM method benefits from the haplotype and the phenotype information, and from the penetrance model. Another benefit is that the EM method does not need many markers, we used only 500 of them. Moreover, it is very interesting to evaluate the sensitivity π^1^ and the specificity π^0^ for the naive method, and to compare them with our previous estimates obtained using our method. This comparison is illustrated in Figure 9B, which shows π^1^ and π^0^ (from Figure 9B), and the same rates for the four most associated markers using the naive method. The EM method (plain lines) shows both increased sensitivity and increased specificity around the location of the TIM, as expected, whereas the naive method has a very high specificity and a very low sensitivity. To complement these results, Figure 9C shows the relation of the sensitivity to 1-specificity, such that the darkness of each of the 7503 points is proportional to −log_10_ *p*-value of the association between the marker and the phenotype; one can see that the most significantly associated markers are not the ones exhibiting the highest sensitivity and specificity. The best marker (regarding sensitivity and specificity) is at the top-left of the figure, and is not near being the most associated one. It is important to note, however, that these results depend of the strength of the genetic model: if RR_1_ = RR_2_ = 10, then it is likely that one of the marker could perform as good or better than the EM method, because the association between the marker and the phenotype in this case would be more direct. This illustrates that our EM method surpasses the naive method in the most interesting cases.

## 5. Conclusion

We have shown how to build an EM algorithm to jointly estimate haplotypes and unknown alleles at the TIM conditionally on the phenotype. In contrast to other methodologies, we use the phenotypic information available, and estimate the frequencies of haplotypes for non carriers, *V*_0_, and for carriers, *V*_1_; the method also estimates the alleles *T* = (δ_1_, δ_2_), opening new avenues. This method to estimate the alleles at the TIM can also be used with resolved haplotypes, and with missing values. We have shown that the methodology is robust to the sampling from case/control design, which is commonly used in genetic studies. We benchmarked the method on data simulated under the coalescent. The efficiency of the method to infer the alleles at the TIM depends mostly on the strength of the genetic model: when the relative risks are high, the success rates of correctly estimating the alleles are high. This implies that it would be relatively easy to infer TIM alleles for mendelian traits, however we probably need more data when relative risks are low. We observed that neither the frequency of the disease nor of or the causal alleles in the population had any impact on the efficiency of the method. This was to be expected given the case/control design, which implies an enrichment in cases, and thus in causal alleles, in the samples. We also compared our methodology to a naive method, which consists of estimating the alleles at the TIM by the alleles of the marker the most significantly associated with the phenotype. By studying specificity and sensitivity, we have shown that the proposed method provides both higher specificity and higher sensitivity, especially around the true position of the TIM.

## Funding

This work was supported by the Natural Sciences and Engineering Research Council of Canada by a grant to the first FL. GB has received scholarships from FQRNT and NSERC.

### Conflict of interest statement

The authors declare that the research was conducted in the absence of any commercial or financial relationships that could be construed as a potential conflict of interest.
